# A new dynamic *in vitro* model for evaluating antimicrobial activity against bacterial biofilms on central venous catheters

**DOI:** 10.1128/spectrum.00237-24

**Published:** 2024-07-26

**Authors:** Liangyan Fang, Yunqian Qiao, Xiuting Li, Changbin Wang, Chunqiao Li, Tongqing Luan, Wenqing Wang

**Affiliations:** 1 Shandong Institute of Medical Device and Pharmaceutical Packaging Inspection, Jinan, Shandong, China; 2 NMPA Key Laboratory for Safety Evaluation of Biomaterials and Medical Devices, Jinan, Shandong, China; University of Debrecen, Debrecen, Hungary

**Keywords:** central venous catheter, bloodstream infection, *in vitro *model, antimicrobial, biofilm inhibition

## Abstract

Central venous catheters (CVCs) are widely used for intravenous medication administration. However, biofilm formation along the catheter surface is the main most important cause of catheter-related bloodstream infections. Nowadays, several antimicrobial-coated catheters are available to prevent biofilm development. In this study, we introduced a new dynamic *in vitro* model to evaluate the antimicrobial activity against bacterial biofilms on CVCs. Rifampicin–minocycline-coated catheters and control catheters without antimicrobial component were assembled into the model to test the antimicrobial activity on external surface and internal surface. After 1 h irrigation of *Staphylococcus epidermidis* or *Staphylococcus aureus* preculture and 23 h irrigation of Trypticase Soy Broth, the viable adherent organism was collected and counted. The enumeration results showed that the number of bacteria attached to antibacterial catheter was significantly less than that of the control catheter, both on external surface (*P* < 0.05) and internal surface (*P* < 0.05). The results were further confirmed by the scanning electron microscopy. In conclusion, the dynamic *in vitro* model can be applied to evaluate the antimicrobial activity against bacterial biofilms grown on the external and internal surfaces of CVCs used in clinical practice.

**IMPORTANCE:**

For the first time, a new dynamic *in vitro* model was constructed to evaluate the antimicrobial activity against bacterial biofilms on central venous catheters (CVCs) on both external surface and internal surface. This model could be applied to evaluate the antimicrobial activity against bacterial biofilms not only on CVCs but also other types of catheters.

## INTRODUCTION

Central venous catheters (CVCs) are widely used for intravenous medication administration, parenteral nutrition, hemodynamics surveillance, and fluid replacement (1). It is widely used in clinical practice, especially in intensive care units (ICUs) (2). However, the utilization of CVCs may result in catheter-related bloodstream infection (CRBSI), leading to significant morbidity and mortality, increased cost for hospitalized patients, and extended hospital stays (3). In China, it is reported that the incidence of CRBSI varied from 1.67 to 16.96 per 1,000 catheter days (4).

Microbial colonization and biofilm formation along catheter surfaces are the main causes of CRBSI. Biofilm is a complex organic material consisting of microorganisms growing in colonies within an extracellular mucopolysaccharide substance, which they produce, allowing the bacteria to be tolerant to antiseptics and antibiotics (5). Microorganisms may colonize both external and luminal surfaces of CVCs, and it is reported that short-term (<10 days) catheters in place are more heavily colonized on the external surfaces while catheters for up to 30 days tend to be more heavily colonized within the lumen (6). Bacteria that colonize CVCs may originate from the skin at the insertion site and migrate along the catheter’s external surface (named external pathway), or originate through hematogenous seeding of the device from another infected nidus in the catheterized patient (named luminal pathway). The most common microorganisms recovered from CVCs include coagulase negative staphylococci, *Staphylococcus aureus*, *Pseudomonas aeruginosa*, *Enterococcus faecalis*, and *Candida* species (7). Nowadays, different antimicrobial-coated catheters have been developed to prevent biofilm development, which can be divided into release-based and contact-based catheters based on their antimicrobial strategies (7).

At present, several approaches have been reported to assess the antimicrobial effect of catheters using *in vitro* models, including static models (6, 8) and dynamic models (9, 10). The static models test the antimicrobial activity against microorganism growing on 96-well polystyrene plates, without reflecting the biofilm conditions on medical devices. Several flow systems have been studied to assess *in vitro* anti-biofilm efficacy by simulating the *in vivo* situations, but most of them have the limitation that the device has to be shaped in a determined form and size for biofilm formation (11, 12). Besides, a dynamic *in vitro* model for evaluating biofilm inhibition activity using clinical-used catheters was reported (9), but the system could only mimic extraluminal biofilm formation. Thus, an *in vitro* model that can mimic luminal biofilm formation is still needed.

The aim of this study was to develop an *in vitro* model that enables the evaluation of the effect of antimicrobial-coated catheters on biofilms on both external and luminal surfaces. The experimental parameters were designed to simulate the colonization of microorganism and biofilm formation on CVCs.

## MATERIALS AND METHODS

### Bacterial strains and culture conditions

*S. aureus* ATCC 6538 and *Staphylococcus epidermidis* ATCC 12228 were used in this study. Frozen stock culture of strain was first inoculated onto Trypticase Soy Agar (TSA, BD, USA) at 35°C for 24 h. Single bacterial colonies were taken from the culture and transferred into Trypticase Soy Broth (TSB, BD, USA) until the mid-exponential phase.

### Catheters

Commercially available antimicrobial CVCs (made of polyurethane, external diameter: 2.1 mm) [Lepu Medical Technology (Beijing) Co., Ltd, China] were tested in the study. Disposable CVCs (made of polyurethane, external diameter: 2.1 mm) (Beijing Target Medical Technologies, Inc, China) with no antimicrobial agent were served as control. According to the manufacturer’s instructions, the antimicrobial CVCs are coated with rifampicin (150 µg/cm) and minocycline hydrochloride (150 µg/cm), both on the external and luminal surfaces. And the manufacturer claims that the loading density per square cm was equal between external and luminal surfaces.

### Zone of inhibition test

The inhibition zone assay protocol was based on Clinical and Laboratory Standards Institute guidelines (13). The zone of inhibition assay was performed for both external and luminal surfaces. For the external surface, the antimicrobial and control catheters were cut into 0.5 cm long segments and vertically embedded in TSA plates, which were swab-inoculated with one of the bacteria (appropriate 10^5^ CFU/plate). The plates were incubated overnight at 30–35°C. Diameters of the inhibition zones (excluding the catheter diameters) were measured to reflect the efficacy of bacterial inhibition. To assay antibiotic leaching from the luminal surface, we cut the catheter segments in half longitudinally and then vertically embedded in TSA plates swab inoculated with bacteria.

### Biofilm reactor model system design

**FIG 1:**
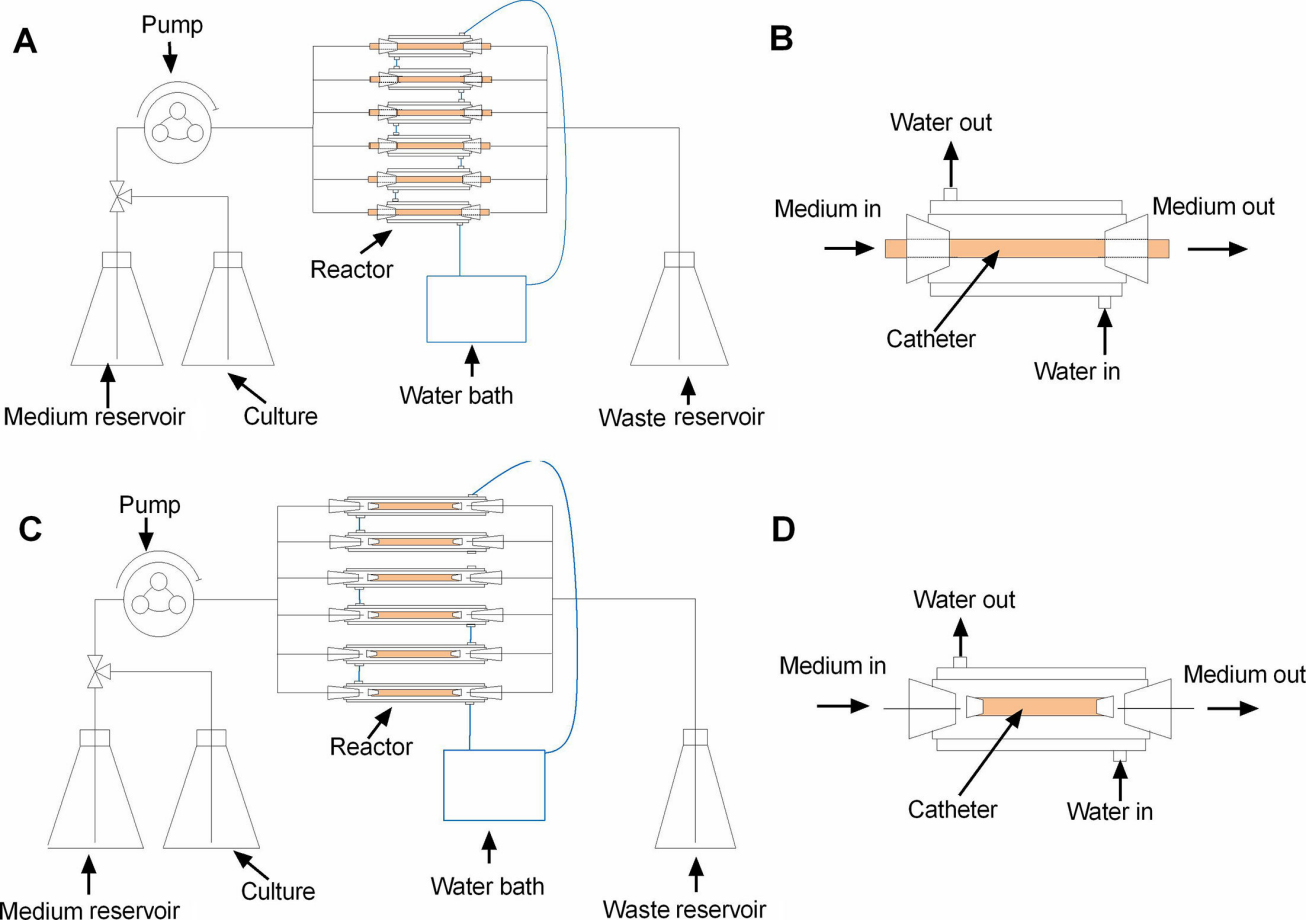
The schematic diagram of biofilm reactor model system for CVCs. The model system for internal (A) and external (C) surface of CVCs. The reactor detail for internal surface (B) and external surface (D) of CVCs.

The *in vitro* biofilm reactor model developed by our group is designed as a dynamic system. The device contains six independent reactors, inside which six catheter segments are implanted independently, three reactors for antimicrobial CVCs and three for control CVCs. For each reactor, a temperature control compartment is set outside the reactor by connecting with a temperature control water circuit. The water flow is parallel to the axis of all samples (catheter segments). This model is designed for extraluminal and intraluminal antimicrobial effect testing, respectively. For intraluminal antimicrobial effect test, the implanted catheter segments are connected with the medium pipeline to allow liquids flow through (Fig. 1A and B). For evaluating the extraluminal antimicrobial effect, catheter segments with both ends sealed with nails are placed in the reactor aseptically and exposed to the flow medium (Fig. 1C and D). The biofilm reactor model is sterilized by high-pressure steam treatment.

### Biofilm formation

The bacterial culture (overnight culture) in TSB was simultaneously pumped through the six reactors in the device at the same speed of 40 mL/h for 1 h. Sterilized TSB medium, which simulates the blood flow was pumped to establish biofilm with a continuous flow of nutrients, at the speed of 20 mL/h for an additional 23 h. The control catheters were used to check normal biofilm formation.

### Enumeration of viable adherent microorganisms

After irrigation by bacterial suspension for 1 h, followed by TSB medium for 23 h, catheter segments were removed from the reactor model system and cut into 1 cm long segments. The catheter segments were washed gently with 20 mL phosphate-buffered saline (PBS, pH 7.2) for three times to remove free and loosely adhering bacteria and then moved to 15 mL sterilized centrifuge tubes prefilled with 5 mL PBS. The high-speed vortex was performed for 100 s, followed by sonication for 5 min at 40 kHz to safely and completely recover adherent cells from catheter segments. While for the bacteria on the luminal surface, the catheter segments were sliced in half longitudinally in order to expose the luminal surface, and then washed with 20 mL PBS for 3 min (9, 14). The bacterial suspension was subjected to suitable gradient dilution with PBS, 1 mL of which was transferred into separate 90 mm Petri dishes. 15–20 mL TSA, cooled to 42–45°C, was then poured into dishes. The agar plates were incubated at 30–35°C for 48 h to count the number of bacterial colonies. The counting results were standardized to the catheter surface area (CFU/cm^2^).

### Scanning electron microscopy

Scanning electron microscopy was performed to visualize the biofilm formation on the surface of catheter segments. The segments removed from the device were fixed with 2.5% glutaraldehyde in sodium cacodylate buffer for 2 h at 4°C, and then dehydrated in a series of aqueous ethanol (30%, 50%, 70%, 90%, and 100%) (8). For the luminal surfaces, we first sliced catheter segments in half longitudinally in order to expose the luminal surface, followed by fixation and dehydration. Biofilm was examined in a SEM-FEG Hitachi SU8010 (Hitachi High-Technologies Europe GmbH; Krefeld, Germany).

### Statistical analysis

Statistical analysis was performed by SPSS software. All testings were performed in triplicate. The data were recorded as mean ± standard deviation. The number of viable microorganisms adhering to the catheters were recorded as CFU/cm^2^ and compared by the *t*-test (*P* < 0.05 was considered statistically significant).

## RESULTS

### Determination of inhibition zones

**FIG 2:**
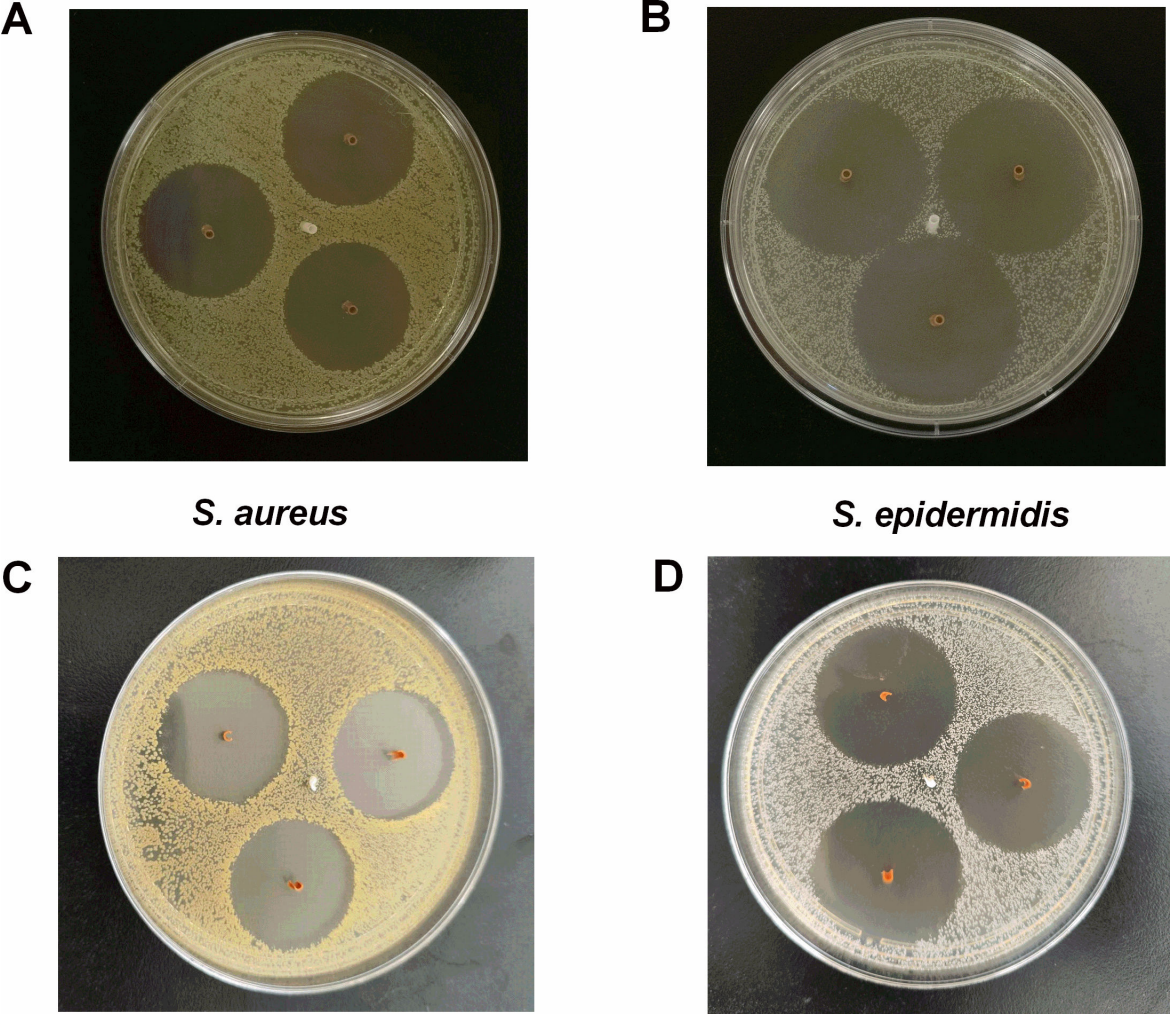
The inhibition zones of CVCs against *S. aureus* (A and C) and *S. epidermidis* (B and D). Orange sample: antibacterial CVC; white sample: control CVC.

Since the loaded antibiotics can be released and diffuse out, the antibacterial activities of CVCs were initially estimated by the agar diffusion assay. For the external surface, the zones of inhibition of each organism were represented in Fig. 2A and B (S. *aureus*) (*S. epidermidis*). The diameter of the inhibition zones was recorded and presented in Table 1. The results showed that, compared to control catheters, the antibacterial catheters could inhibit the growth of *S. aureus* and *S. epidermidis*on agar plates. For the luminal surface, we cut the catheter segments in half longitudinally, and then vertically embedded in TSA plates premixed with bacteria as presented in Fig. 2C and D (S. *aureus*) (*S. epidermidis*). We found that the zone of inhibition was still a complete circle, indicating that the inhibition zones of the luminal surface were consistent with that on the external surface.

**TABLE 1:** The diameter of zones of inhibition

Organism	Sample	Diameter of zone of inhibition (mean^*a*^± standard deviation, mm)
*S. aureus*	Control CVC	None
	Antibacterial CVC	26 ± 0.53
*S. epidermidis*	Control CVC	None
	Antibacterial CVC	33 ± 0.49

^*a*^ Mean of the zones of three segments.

### Extraluminal antimicrobial effect test

**FIG 3:**
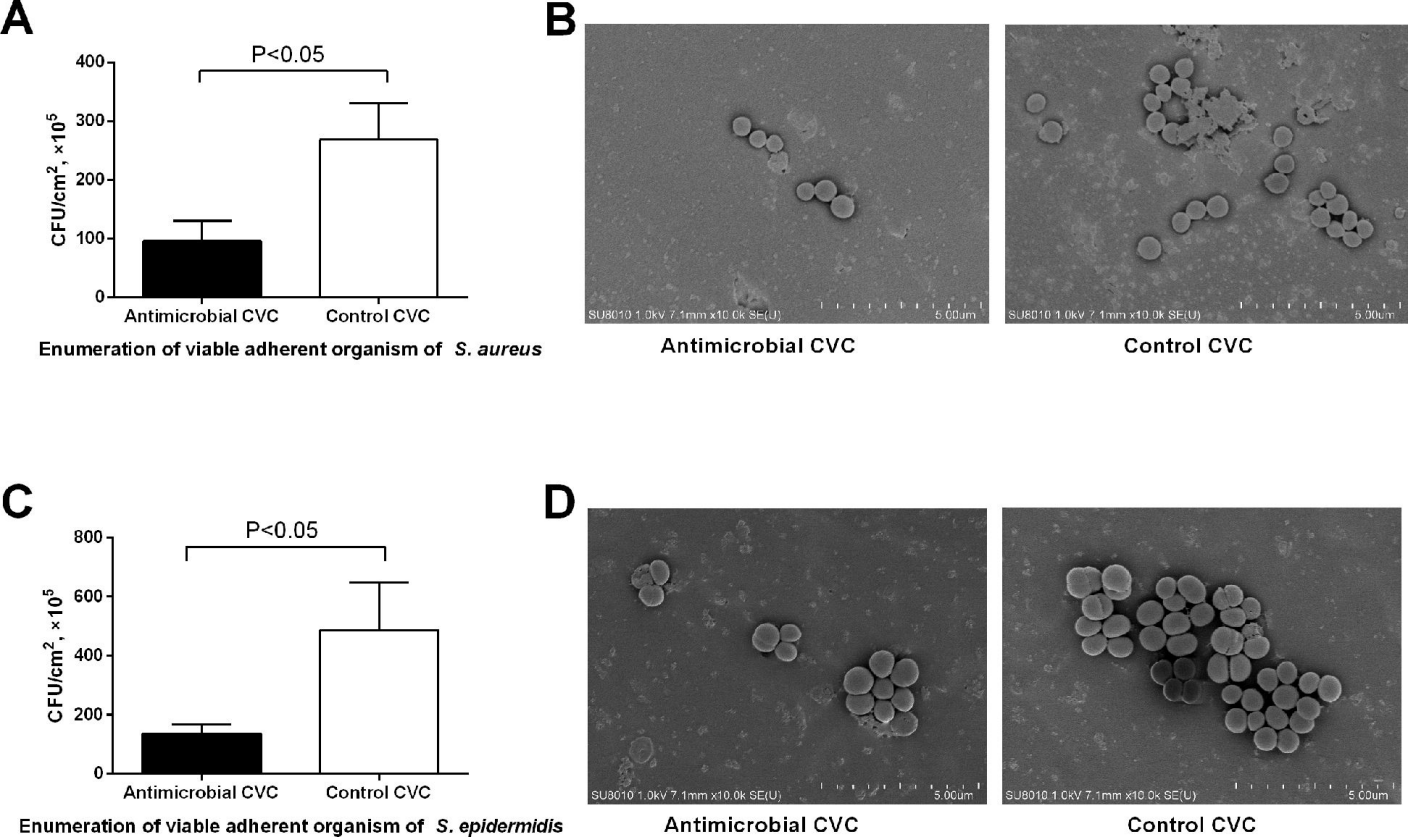
Extraluminal antimicrobial activities of CVCs. The comparative number of *S. aureus* (A) and *S. epidermidis* (C) attached to external surfaces of catheters. The representative scanning electronic micrograph of *S. aureus* (B) and *S. epidermidis* (D) biofilm formation on CVCs.

In order to validate the antimicrobial effect of CVCs using the *in vitro* model built in this study, extraluminal activity was first tested. The catheters sealed with nails were placed in the reactor and exposed to the flow medium. After 24 h of perfusion, the adherent bacteria represented the biofilm attached to and colonized on the external surface. The viable adhered cells were recovered by validated vortexing and sonication method, and quantified by agar plating. As shown in Fig. 3A, the number of *S. aureus* attached to the external surface of antibacterial CVCs (9.5 × 10^6^ ± 2.9 × 10^6^ CFU/cm^2^) was significantly less than that of the control catheters (2.7 × 10^7^ ± 5.0 × 10^6^ CFU/cm^2^) (*P* < 0.05). As for *S. epidermidis*, a significant difference was also presented between the number of bacteria attached to antibacterial catheter surfaces (1.3 × 10^7^ ± 2.7 × 10^6^ CFU/cm^2^) and that of the control catheters (4.9 × 10^7^ ± 1.4 × 10^7^ CFU/cm^2^) (*P* < 0.05) (Fig. 3C). Furthermore, the SEM was performed to observe the bacterial adhesion of the samples after 24 h of co-incubation with *S. aureus* (Fig. 3B) and *S. epidermidis* (Fig. 3D). There was less bacterial adhesion on the surface of anti-microbial CVC samples compared with control CVC for both *S. aureus* and *S. epidermidis*. The SEM results were consistent with the zones of inhibition and colony counting results.

### Intraluminal antimicrobial effect test

**FIG 4:**
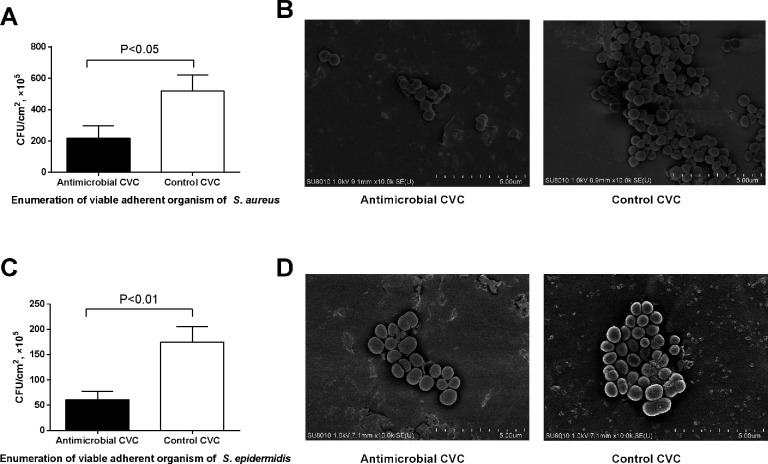
Intraluminal antimicrobial effect test. The comparative number of *S. aureus* (A) and *S. epidermidis* (C) attached to external surfaces of catheters. The representative scanning electronic micrograph of *S. aureus* (B) and *S. epidermidis* (D) biofilm formation on CVCs.

For intraluminal antimicrobial effect test, the implanted catheter segments were connected with the medium pipeline. After removed from the reactor, 1 cm segments were cut in half longitudinally. For *S. aureus*, the count of bacteria attached to the inner surface of antibacterial catheters (2.2 × 10^7^ ± 6.6 × 10^6^ CFU/cm^2^) was significantly reduced than that of control catheters (5.2 × 10^7^ ± 8.2 × 10^6^ CFU/cm^2^) (*P* < 0.05) (Fig. 4A). Similarly, the number of *S. epidermidis* of antibacterial catheter (6.1 × 10^6^ ± 1.3 × 10^6^ CFU/cm^2^) was significantly decreased relative to the number of bacteria attached to control catheter surface (1.8 × 10^7^ ± 2.5 × 10^6^ CFU/cm^2^) (*P* < 0.05) (Fig. 4C). The SEM was performed to observe the bacterial adhesion of the samples after 24 h of co-incubation with *S. aureus* (Fig. 4B) and *S. epidermidis* (Fig. 4D). There was less bacterial adhesion on the surface of anti-microbial CVC samples compared with control CVC for both *S. aureus* and *S. epidermidis*. The SEM results were consistent with the colony counting results.

## DISCUSSION

Nowadays, CRBSI is a significant public health problem and has an associated economic impact. In response to the increased prevalence of catheter-related infections, different strategies have been used to protect catheter surfaces from microbial colonization and biofilm development. These include physical/chemical modifications of catheter surfaces (7) and coating/impregnating catheters with antimicrobial agents, such as antibiotics, antiseptic, silver, or chitosan (15–17). However, due to the high risk of creating microbial resistance to antimicrobial treatments, the US Food and Drug Administration (18) discourages the abuse of antimicrobial agents on devices unless a strong rationale is provided to demonstrate the clinical benefit outweighs the associated risk (18). FDA recommends medical device manufacturers provide detailed information and pre-clinical testing to verify the safety and efficacy of devices that include antimicrobial agents (18). Thus, in order to promote the proper development and evaluation of the antimicrobial efficacy of medical devices, appropriate *in vitro* testing tools are needed to mimic the clinical settings and analyze the performance of devices before performing delicate and expensive *in vivo* studies. With regard to antimicrobial CVC, despite intensive research in biofilm preventive strategies, there is only a limited choice of *in vitro* evaluation models.

At present, there is no universally accepted *in vitro* model to reproduce the biofilm formation on catheter. Curtin *et al.* developed an *in vitro* model to mimic biofilm formation on luminal surface based on a modified drip flow reactor (14). And Garcia *et al.* designed a device (called the *Sevilla* device) that enables antimicrobial activity against bacterial biofilm to be evaluated on the external surface of intravascular catheter (9). In this study, we developed a well-established *in vitro* model. With the use of this model, we evaluated the antibacterial effect of commercial minocycline–rifampicin coating CVCs on both external surface and luminal surface with continuous culture supply.

In the last two decades, CVCs coated with conjugated minocycline and rifampicin have been widely used because of their additive properties (7, 19). Rifampin inhibits RNA synthesis in bacteria by binding to and inhibiting bacterial RNA polymerase (20), and minocycline inhibits bacterial protein synthesis by preventing the association of tRNA with the bacterial ribosome (21). Both of them are highly potent against key Gram-positive pathogens and some Gram-negative pathogens, but have less potency against other Gram-negative pathogens and fungal pathogens (22). In the present study, minocycline−rifampicin-impregnated CVCs were applied to detect the antibacterial activity.

CVC infections are mainly due to coagulase negative staphylococci, *S. aureus*, *Klebsiella pneumoniae*, *P. aeruginosa*, *E. faecalis*, and *Candida* species (7). In this study, *S. epidermidis* and *S. aureus* were selected to detect the biofilm inhibition effect of antimicrobial CVCs. The antibacterial catheters presented a powerful inhibition effect on the *S. aureus* and *S. epidermidis* plates. And the enumeration of viable adherent organisms on antimicrobial catheters was significantly less than that on control catheters (*P < 0.05*) for both external and luminal surfaces. These results were in accordance with the research that these two antibiotics are powerful agents against Gram-positive pathogens.

Still, there are some limitations in this study. More work is needed to examine the antimicrobial effect on Gram-negative pathogens such as *K. pneumoniae* and *P. aeruginosa*. Besides, further work is needed to evaluate the antimicrobial effect for a longer period of time that correlated with the *in vivo* situation using this model. CVCs with different mechanisms of action will be tested using this model to validate its wide applicability.

In conclusion, we successfully designed a dynamic *in vitro* model to mimic biofilm formation on CVCs. Using this model, the antimicrobial effect was examined on both external and luminal surfaces of minocycline–rifampicin-coated catheters.
